# Exploring perceptions, awareness, and healthcare-seeking behaviors related to muscle weakness among Saudi adults: a cross-sectional study

**DOI:** 10.1186/s12889-026-28692-4

**Published:** 2026-08-03

**Authors:** Ahmed M. Ashour, Ali M. Alshahrani, Bader A. Alhazmi, Aliah Alhayyan, Rawan Alhayyan

**Affiliations:** 1 Department of Pharmacology and Toxicology, College of Pharmacy, Umm Al-Qura University, Makkah, Saudi Arabia; 2 King Salman Center for Disability Research, Riyadh, 11614 Saudi Arabia; 3 Department of Clinical Pharmacy, College of Pharmacy, Taif University, Taif, Saudi Arabia; 4 Third Riyadh Health Cluster, Huraymila General Hospital, Riyadh, Saudi Arabia; 5 Family Medicine Department, Prince Sultan Military Medical City, Riyadh, Saudi Arabia

**Keywords:** Muscle weakness, Awareness, Myasthenia gravis, And perceptions

## Abstract

**Background:**

Muscle weakness is a common health concern that can significantly affect quality of life. Understanding public awareness, perceptions, and healthcare-seeking behaviors is important for early detection and management. This study aimed to assess awareness, perceptions, and healthcare-seeking behaviors related to muscle weakness among Saudi adults.

**Methods:**

A cross-sectional survey was conducted among 1,000 adults in Saudi Arabia between August and October 2025. A structured questionnaire assessed knowledge of muscle weakness, perceived causes and symptoms, attitudes toward treatment, and healthcare-seeking behaviors. Descriptive statistics and chi-square tests were used to examine associations between demographic factors and awareness-related outcomes.

**Results:**

A total of 1,000 participants completed the survey. Only 27.8% reported having heard of muscle weakness as a medical condition. Perceptions of susceptibility and severity as well as consequences showed considerable variability with uncertainty evident across most domains. Attitudes toward seeking medical advice and adopting preventive behaviors were mixed, with many participants expressing uncertainty or low likelihood of action. Although some respondents reported willingness to encourage others to seek care and to make lifestyle changes, a substantial proportion remained hesitant or unsure. Approximately one-third of participants reported personal or indirect experience with muscle weakness with impacts on daily life included increased dependence and frequent healthcare utilization. Statistically significant differences were observed in the distribution of responses across knowledge, perception, attitude, and experience domains (*p* < 0.05 in selected items).

**Conclusions:**

Among Saudi adults, there is substantial variability in perceptions, attitudes, and personal or indirect experience on muscle weakness as a medical condition. There are persistent gaps in health literacy and uncertainty regarding symptom recognition and severity appraisal or appropriate healthcare-seeking behavior. Accordingly, there is a need for targeted public health interventions to improve awareness and promote early recognition of muscle weakness through primary care services and trusted digital platforms.

**Supplementary Information:**

The online version contains supplementary material available at https://doi.org/10.1186/s12889-026-28692-4.

## Introduction

Muscle weakness which is defined as a significant reduction in muscle strength or an inability to exert normal force can profoundly impact the quality of life and daily functioning [33]. This common clinical manifestation can arise from a variety of causes from neurological disorders to chronic diseases and malnutrition as well as lifestyle factors like physical inactivity or poor nutrition [25]. The condition is particularly concerning in neurological disorders where weakness is frequently a primary symptom. Among these, myasthenia gravis (MG) stands out as a major cause of progressive muscle weakness. This condition is characterized by dysfunction at the neuromuscular junction leading to muscle weakness that worsens with activity and improves with rest [20]. MG which is notably a chronic autoimmune condition affects skeletal muscles and can impair vital functions such as breathing and swallowing if not properly managed [14]. Major organizations, including the World Health Organization (WHO) and the European Working Group on Sarcopenia in Older People (EWGSOP2), increasingly recognize muscle weakness as an important public health concern because it contributes to impaired mobility, loss of independence, reduced quality of life, increased hospitalization, and premature mortality, particularly among older adults and individuals with chronic diseases [13]. The WHO identifies the preservation of intrinsic capacity, including musculoskeletal function, as a central component of healthy ageing [30]. Likewise, the EWGSOP2 recognizes low muscle strength as the principal indicator of probable sarcopenia and recommends early identification to prevent disability and functional decline [13]. Similarly, evidence-based guidance from the American Academy of Neurology (AAN) emphasizes the importance of early evaluation of persistent or progressive muscle weakness because it may indicate an underlying neuromuscular disorder requiring timely diagnosis and management [22]. This recommendation is consistent with more recent clinical guidance for the diagnosis and management of acute neuromuscular weakness [29].

In addition to neurological disorders, muscle weakness can result from chronic diseases such as diabetes, in which peripheral neuropathy leads to muscle atrophy and weakness [16], or from conditions like cancer and rheumatoid arthritis, both of which can significantly affect muscle strength [24]. Malnutrition and vitamin deficiencies, especially in older adults, also contribute to muscle fatigue and frailty [37]. Moreover, sedentary lifestyles and aging are well-documented risk factors for muscle weakness, with physical inactivity leading to muscle atrophy and functional decline [10].

The importance of muscle weakness is particularly relevant in Saudi Arabia which is a context where several major risk factors are highly prevalent. National studies have consistently reported high levels of physical inactivity among adults, alongside increasing rates of obesity and diabetes mellitus, all of which are associated with impaired muscle function, reduced physical performance, and accelerated functional decline [4, 5]. In addition, Saudi Arabia is experiencing demographic ageing, with the proportion of older adults expected to increase substantially over the coming decades, further increasing the burden of conditions associated with muscle weakness [9]. These trends highlight the need for greater public awareness regarding the recognition, prevention, and timely management of muscle weakness.

Although muscle weakness is a common manifestation of numerous neurological, metabolic, and age-related disorders, public awareness remains limited in many countries. Studies have shown that many individuals attribute progressive weakness, fatigue, or declining physical performance to normal ageing rather than recognizing these symptoms as potential indicators of underlying disease [35]. Such misconceptions may delay healthcare seeking, contribute to late diagnosis, and reduce opportunities for timely intervention. Educational attainment, health literacy, and access to reliable health information have consistently been associated with greater awareness and earlier healthcare utilization, highlighting the importance of public education initiatives [31]. Besides, several studies have addressed the epidemiology and societal implications of muscle weakness. According to Smith et al. [32, 34], public awareness of neuromuscular diseases is generally low which is a factor linked to delays in diagnosis and treatment. In another study, Jones et al. [18, 19] highlighted the role of education and community outreach in improving early recognition of muscle-related disorders. Fatigue and diminished physical ability which are vital common symptoms are frequently mistaken for normal aging [11, 12]. Moreover, cultural stigmas and misinformation about conditions like conditions around muscle weakness further exacerbate underreporting, particularly in underrepresented groups all of which can be explained within the context of the Health Belief Model (HBM) [1, 2, 17]. In a study by O’Neill et al. [27, 28], the importance of guidance from healthcare professionals was underscored, with over 70% of participants relying on primary care physicians for disease management. However, only 40% could accurately identify symptoms related to muscle weakness, indicating a gap in public health education.

Despite the widespread impact of muscle weakness, there is a notable gap in public awareness regarding its causes, early signs, and effective management strategies. Research indicates that while higher education levels are positively correlated with a better understanding of muscle weakness and its implications, awareness remains low among the general population, especially in rural and underprivileged areas [33]. This lack of awareness can delay diagnosis and treatment, leading to poorer outcomes [25]. Furthermore, misconceptions about the causes and symptoms of muscle weakness, particularly in conditions such as myasthenia gravis, often lead to misdiagnosis and inappropriate treatment [16]. Most notably from this is that despite the growing recognition of muscle weakness as an important clinical and public health concern the published evidence describing public awareness remains limited, particularly in Saudi Arabia. Most published of the published studies have focused on the clinical management of specific diseases or patient populations rather than evaluating how members of the general public recognize muscle weakness and understand its causes, perceive its seriousness and decide when to seek medical care. Furthermore, few studies from Saudi Arabia or the Gulf region have comprehensively examined public knowledge, attitudes, misconceptions, personal experiences, and healthcare-seeking behaviors related to muscle weakness. This lack of evidence limits the development of targeted educational strategies and public health interventions aimed at promoting earlier recognition and appropriate healthcare utilization.

Given the complex, multifactorial nature of muscle weakness, it is essential to assess how different demographic groups perceive the condition and which factors influence their awareness, perceptions, and healthcare-seeking behaviors. For instance, studies show that younger individuals are more likely to seek medical consultation for muscle weakness than older adults, who may attribute their symptoms to aging [10]. Furthermore, gender and education level significantly affect both the awareness of the condition and the likelihood of pursuing appropriate healthcare [37].

### Main objectives of the study

In view of these knowledge gaps, in the current study our primary focus was to evaluate public awareness, perceptions, attitudes, personal experiences, and healthcare-seeking behaviors related to muscle weakness among adults in Saudi Arabia. Specifically, we assessed knowledge of the causes, symptoms, and management of muscle weakness; explored awareness of underlying medical conditions associated with muscle weakness; examined perceived impacts on lifestyle and physical activity; and identified factors associated with healthcare-seeking behavior. This objective was driven by the consideration that through providing population-based evidence from Saudi Arabia, the findings are important as they can inform future public health education initiatives, promote earlier recognition of muscle weakness, and support strategies for timely diagnosis and intervention. Also, an understanding of these factors can shed light on barriers to early intervention and may guide public health campaigns as well as that understanding public knowledge and healthcare-seeking behaviors may also help inform future educational interventions and early diagnosis strategies.

## Methodology

### Study design

A cross-sectional online survey was conducted to assess awareness, perceptions, and healthcare-seeking behaviors related to muscle weakness among Saudi adults. A structured survey questionnaire was used to collect data on participants’ awareness and attitudes toward muscle weakness from August to October 2025.

### Study population

The study targeted adults aged 18 years and above residing in various regions of Saudi Arabia. This broad inclusion criterion was used to capture a diverse representation of the Saudi adult population. The final sample comprised 1,000 participants, considered adequate for descriptive and exploratory analyses of awareness, perceptions, and healthcare-seeking behaviors related to muscle weakness among Saudi adults. However, because convenience sampling was used, the findings should be interpreted with caution and may not be fully generalizable to the entire adult population in Saudi Arabia. The inclusion criteria were being aged 18 years or older, the ability to understand and respond to the survey questions in Arabic, and residence in Saudi Arabia. Participants from various age groups, educational backgrounds, and employment statuses were included to ensure diversity. The exclusion criteria were individuals under 18 years of age and participants unable to read or understand the survey questions.

### Sampling method and sample size calculation

A convenience sampling approach was used to recruit participants through online platforms. The survey was distributed to adults across different age groups, genders, educational levels, and geographic regions in Saudi Arabia to support demographic diversity. This approach allowed the study to explore variations in awareness, perceptions, and healthcare-seeking behaviors related to muscle weakness across different participant groups. The minimum sample size was calculated a priori using Cochran’s formula for cross-sectional studies where we assumed a 95% confidence level and 5% margin of error with a conservative estimated proportion of 0.5 to maximize sample size. An additional allowance of 10% of the determined sample size was added to account for potential non-response and incomplete questionnaires.

### Data collection

Data were collected using an online questionnaire developed specifically for this study. The questionnaire had not been previously published elsewhere. An English-language version of the questionnaire has been uploaded as Supplementary File 1 and cited in the manuscript. The questionnaire consisted of multiple sections. The first section collected demographic data, including age, gender, education level, occupation, and region of residence. The second section assessed participants’ knowledge of muscle weakness, including causes, symptoms, and potential complications. The third section explored perceptions and attitudes toward muscle weakness, including perceived susceptibility, perceived severity, treatment-seeking attitudes, and healthcare-seeking behavior. The questionnaire explored common misconceptions and factual knowledge about the underlying causes of muscle weakness. It also assessed participants’ perceptions and attitudes toward muscle weakness, including perceived susceptibility, perceived severity, attitudes toward seeking treatment, and healthcare-seeking behavior. Participants were also asked whether they would consult a healthcare provider for muscle weakness or consider such symptoms part of normal aging.

The questionnaire used Likert-scale responses to quantify attitudes and knowledge levels: 0 = strongly disagree/not confident/not important; 1 = disagree/slightly confident/somewhat important; 2 = agree/confident/important; and 3 = strongly agree/very confident/very important. In all other Likert scales, the same scaling was used so that for five scales the lower value was 0 and the upper value for five scales was 5. The survey was distributed through various online platforms, including social media, community organizations, and healthcare facilities, to ensure broad outreach and diverse participation across different sectors of the population. Efforts were made to reach individuals in rural areas and those with lower access to digital resources. The questionnaire was pretested with 10% of the intended sample size to assess clarity, readability, and face validity. Based on the feedback received, minor adjustments were made to the wording and format before final distribution. The English-language version of the questionnaire is provided as Supplementary File 1.

### Data analysis

Descriptive and inferential statistics were used to analyze the data. Frequencies and percentages were used to summarize categorical variables, while means and standard deviations were used for continuous or ordinal variables where appropriate. Chi-square tests were used to examine associations between demographic characteristics and awareness, perceptions, and attitudes toward muscle weakness. Statistical significance was set at *p* < 0.05. All analyses were conducted using IBM SPSS Statistics version 29.

### Ethical considerations

The study adhered to ethical guidelines for research involving human participants. Informed consent was obtained from all participants before data collection. Participants were informed of the study objectives, procedures, potential risks, confidentiality measures, and their right to withdraw. Data were anonymized to protect participant privacy. The study protocol was reviewed and approved by the Institutional Review Board at Umm Al-Qura University before data collection commenced, approval number HAPO-02-K-012–2025-01–2444.

### Significance of the study

This study assessed variations in awareness and perceptions of muscle weakness among different demographic groups in Saudi Arabia. The findings identify knowledge gaps and misconceptions about muscle weakness, its causes, and treatment options, which may guide targeted educational interventions. The study also explored healthcare-seeking behaviors and potential barriers to early diagnosis and treatment. Its significance lies in informing culturally relevant health promotion strategies that support early recognition of muscle weakness and proactive healthcare-seeking behaviors. Additionally, the findings may assist healthcare planners and public health professionals in designing educational initiatives. More particularly in context of the Saudi Health goals, these contributions align with Saudi Arabia’s Vision 2030 healthcare transformation agenda which prioritizes preventive care and health literacy as well as early intervention to reduce the burden of non-communicable and chronic conditions. Strengthening public awareness of functional health conditions such as muscle weakness supports the national goal of building a more proactive and efficient healthcare system.

## Results

### Descriptive statistics of participant profiles and symptom data

The descriptive statistics (Table 1) provide a clear snapshot of the participant demographics and their perspectives on muscle weakness. A greater proportion of the participants were females, 70.3% (*n* = 703), compared to males, 29.7% (*n* = 297), in the sample. Age-wise, the study predominantly included younger participants, with 60.0% (*n* = 600) aged 18 to 49 years. The lower representation of older age groups, especially those aged 30–39 (22.3%) and 40–49 (28.3%), indicates that the study featured a more youthful population, potentially due to the online survey distribution method used. 15.1% (*n* = 151) had post graduate education. Another 22.7% (*n* = 227) having at least a Bachelor's degree level of education, and 25.0% (*n* = 250) indicated to have attained a Diploma level of education. A considerably small proportion of the study participants 27.8% (*n* = 278) reported awareness of muscle weakness. About 8% of the participants indicated to understand chronic conditions like diabetes, heart disease, and malnutrition as being among key causes of muscle weakness. Nevertheless, the 16.7% of respondents who were unaware point to an opportunity for further education and awareness initiatives. Participants exhibited a positive attitude towards seeking medical consultation, with a high mean score (2.5/3) indicating that most people considered it important to consult a healthcare professional for muscle weakness. However, their confidence in identifying symptoms was moderate (mean score of 2.1). Symptom severity, as reported by participants, was moderate, with a mean frequency score of 6.2 out of 10. Additionally, the mean symptom duration of 4.5 days (Table 1).

**Table 1:** Descriptive statistics of participant profiles and symptom data

Variable	Category	Frequency (n)	Percentage (%)	Mean	Standard Deviation (SD)
Gender	Male	297	29.7%	-	-
	Female	703	70.3%	-	-
Age Group	18–29	94	9.4%	-	-
	30–39	223	22.3%	-	-
	40–49	283	28.3%	-	-
	Above 50	400	40.0%	-	-
Awareness of Muscle Weakness	Yes	278	27.8%	-	-
	No	722	72.2%	-	-
Attitudes Towards Treatment	Importance of Consultation (1–3)	-	-	2.5	0.8
	Confidence in Identifying Symptoms (1–3)	-	-	2.1	0.7
Symptoms Severity	Frequency of Symptoms (0–10)	-	-	6.2	2.3
	Duration of Symptoms (1–7 days)	-	-	4.5	1.5

Frequency (N), The number of participants. Percentage (%), The proportion of participants to the total sample size

### Statistical significance of factors influencing muscle weakness awareness

Gender was significantly associated with awareness of muscle weakness (*p* = 0.01), while age was not significantly associated with awareness (*p* = 0.12). Education showed a borderline association with awareness (*p* = 0.05). Region and occupation were not significantly associated with awareness, with *p*-values of 0.10 and 0.08, respectively.

### Knowledge of muscle weakness and associated factors

The analysis of participants’ knowledge about muscle weakness (Table 2) revealed notable findings across several dimensions. When asked whether they had ever heard of muscle weakness as a medical condition, the majority of respondents (72.2%, *n* = 722) reported that they had not heard of it with only 27.8% (*n* = 278). The chi-square analysis showed a statistically significant difference in responses (*p* < 0.01. Regarding the causes of muscle weakness, aging was the most frequently identified cause (37.6%, *n* = 376), followed by chronic diseases such as diabetes and heart disease (16.5%, *n* = 205), lack of physical activity (14.7%, *n* = 147), injuries or trauma (14.5%, *n* = 145), and malnutrition (12.7%, *n* = 127). The chi-square test was not statistically significant (*p* = 0.10).

**Table 2:** Analysis of muscle weakness knowledge

Knowledge Question	Response Option	Frequency (N)	Percentage (%)	Chi-Square (*p*-value)
Have you ever heard of muscle weakness as a medical condition?	No	722	72.2%	< 0.01
	Yes	278	27.8%	
Which of the following do you think are common causes of muscle weakness?	Aging	376	37.6%	0.10
	Chronic diseases	205	16.5%	
	Lack of physical activity	147	14.7%	
	Malnutrition	127	12.7%	
	Injuries or trauma	145	14.5%	
What do you think are common symptoms of muscle weakness?	Muscle fatigue after minimal effort	357	35.7%	0.04
	Difficulty in moving or lifting objects	241	24.1%	
	Reduced ability to perform daily tasks	218	21.8%	
	Pain or discomfort in muscles	139	13.9%	
	Frequent falls or instability	45	4.5%	
Do you believe muscle weakness is a treatable condition?	No	374	37.4%	0.02
	Yes	255	25.5%	
	Unsure	371	37.1%	
Which treatments do you think could be effective for managing muscle weakness?	Medications	256	25.6%	0.15
	Surgery	250	25.0%	
	Physical therapy/exercise	184	18.4%	
	Nutritional/Dietary supplements	184	18.4%	
	Lifestyle modifications	126	12.6%	

(Knowledge Question), the specific questions regarding participants’ knowledge about muscle weakness. (Response Option). The possible answers for each question. (N), number of participants. (%), the proportion of respondents who selected each answer in relation to the total number of respondents. *p*-value is < 0.05, indicates that there is a statistically significant association between the demographic variable and knowledge of muscle weakness; *p*-value is > 0.05, indicates that there is no significant association

Concerning symptoms of muscle weakness, muscle fatigue after minimal effort was the most commonly recognized symptom (35.7%, *n* = 357), followed by difficulty in moving or lifting objects (24.1%, *n* = 241), reduced ability to perform daily tasks (21.8%, *n* = 218), pain or discomfort in muscles (13.9%, *n* = 139), and frequent falls or instability (4.5%, *n* = 45). The chi-square analysis indicated a statistically significant difference in symptom recognition (*p* = 0.04). When asked whether muscle weakness is a treatable condition, 37.4% (*n* = 374) responded "No," 37.1% (*n* = 371) were unsure, and only 25.5% (n = 255) believed that the condition is treatable. The chi-square test demonstrated a statistically significant difference in responses (*p* = 0.02).

With respect to treatment options, 25.6% (*n* = 256) of the participants indicated medications as the most preferred approach, with a closely matching proportion of 25% indicating surgery to be the best way to treat muscle weakness. Physical therapy and dietary supplements each had a preference of 18.4% (*n* = 184) among the participants as the best approach to treat muscle weakness (40.0%, *n* = 400), and lifestyle modification had a frequency of 12.6% (*n* = 126). The chi-square analysis was not statistically significant (*p* = 0.15).

### Analysis of participants’ perceptions and awareness of muscle weakness

The findings showed mixed perceptions regarding personal susceptibility to muscle weakness and its severity as well as its impact on daily life. Regarding the likelihood of developing muscle weakness at some point in their lives, the largest proportion of respondents considered it unlikely (35.0%, *n* = 350) followed by neutral responses (20.1%, *n* = 201). Smaller proportions reported that they were not vulnerable at all (17.3%, *n* = 173), likely (13.9%, *n* = 139), or very likely (13.7%, *n* = 137) to develop muscle weakness. Chi-square test showed no significant differences in the distribution (*p* = 0.08). Regarding the severity of muscle weakness, 28.3% (*n* = 283) perceived it as very severe, while 28.0% (*n* = 280) considered it a low-risk condition and 24.6% (*n* = 246) viewed it as moderately severe (Table 3). A smaller proportion (19.1%, *n* = 191) considered it not severe at all, and no respondents selected the extremely severe category. The chi-square test indicated a statistically significant difference in response distribution (*p* = 0.02).

**Table 3:** Participants’ perceptions regarding muscle weakness

Perception Question	Response Option	Frequency (N)	Percentage (%)	Chi-Square (p-value)
How likely do you think you are to develop muscle weakness at some point in your life?	Not unbearable at all	173	17.3%	0.08
	Unlikely	350	35.0%	
	Neutral	201	20.1%	
	Likely	139	13.9%	
	Very likely	137	13.7%	
How severe do you think the impact of muscle weakness can be on a person’s life?	Not severe at all	191	19.1%	0.02
	Low risk	280	28.0%	
	Moderately severe	246	24.6%	
	Very severe	283	28.3%	
	Extremely severe	0	0.0%	
In your opinion, what is the most challenging aspect of living with muscle weakness?	Physical limitations	233	23.3%	0.10
	Pain and discomfort	306	30.6%	
	Reduced independence	224	22.4%	
	Psychological impact	156	15.6%	
	Social isolation	81	8.1%	
Do you believe muscle weakness only affects older adults?	Yes	264	26.4%	0.11
	No	362	36.2%	
	Not sure	374	37.4%	
How confident are you that you can recognize the signs of muscle weakness in yourself or others?	Not confident	166	16.6%	0.05
	Slightly confident	252	25.2%	
	Somewhat confident	292	29.2%	
	Very confident	290	29.0%	
	Extremely confident	0	0.0%	

Perception Question, specific questions related to how participants perceive muscle weakness and its impact on life. Response Option, The possible answers. Frequency (N), the number of participants who selected each answer. Percentage (%), the proportion of participants who selected each answer

*p*-value is < 0.05, indicates a significant association between the perception responses and demographic variables. *p*-value is > 0.05, no significant difference

When asked about the most challenging aspect of living with muscle weakness, pain and discomfort was the most frequently identified challenge (30.6%, *n* = 306), followed by physical limitations (23.3%, *n* = 233), reduced independence (22.4%, *n* = 224), psychological impact (15.6%, *n* = 156), and social isolation (8.1%, *n* = 81). The chi-square analysis was not statistically significant (*p* = 0.10). Concerning the belief that muscle weakness only affects older adults, 37.4% (*n* = 374) were unsure, 36.2% (*n* = 362) disagreed, and 26.4% (*n* = 264) agreed with the statement. The chi-square test was not statistically significant (*p* = 0.11). With respect to confidence in recognizing signs of muscle weakness, 29.2% (*n* = 292) reported being somewhat confident, while 29.0% (*n* = 290) indicated being very confident. Smaller proportions reported being slightly confident (25.2%, *n* = 252) or not confident (16.6%, *n* = 166), and no respondents selected the extremely confident category. The chi-square analysis showed a borderline statistically significant difference in responses (*p* = 0.05).

### Participants’ attitudes towards muscle weakness and treatment approaches

Table 4 presents participants’ attitudes toward muscle weakness and its management. On the importance of seeking medical advice for symptoms of muscle weakness, the largest proportion of respondents rated it as having low importance (30.4%, *n* = 304), followed by very important (28.7%, *n* = 287) and not important (24.1%, *n* = 241). Only 16.8% (*n* = 168) considered it somewhat important, while no participants showed it as extremely important. The chi-square analysis indicated a statistically significant difference in response distribution (*p* = 0.03). Regarding the likelihood of encouraging someone with muscle weakness symptoms to seek medical help, 25.9% (*n* = 259) reported being unlikely to do so, while 22.0% (*n* = 220) expressed a neutral position. Smaller proportions indicated that they were very unlikely (21.7%, *n* = 217), likely (17.6%, *n* = 176), or very likely (12.8%, *n* = 128) to encourage medical consultation. The chi-square analysis demonstrated a borderline statistically significant difference in responses (*p* = 0.05).

**Table 4:** Participants’ attitudes towards muscle weakness and treatment

Attitude Question	Response Option	Frequency (N)	Percentage (%)	Chi-Square (*p*-value)
How important do you think it is to seek medical advice for symptoms of muscle weakness?	Not important	241	24.1%	0.03
	Low importance	304	30.4%	
	Somewhat important	168	16.8%	
	Very important	287	28.7%	
	Extremely important	0	0.0%	
How likely are you to encourage someone with muscle weakness symptoms to seek medical help?	Very unlikely	217	21.7%	0.05
	Unlikely	259	25.9%	
	Neutral	220	22.0%	
	Likely	176	17.6%	
	Very likely	128	12.8%	
What kind of healthcare professional would you first approach for muscle weakness symptoms?	General practitioner	209	20.9%	0.07
	Physiotherapist	267	26.7%	
	Neurologist	276	27.6%	
	None	248	24.8%	
Would you be willing to make lifestyle changes (e.g., increased exercise, diet modification) to prevent or reduce muscle weakness?	Yes	288	28.8%	0.04
	No	321	32.1%	
	Not sure	391	39.1%	
What sources do you trust the most for information on muscle weakness?	Healthcare providers	157	15.7%	0.09
	Internet/online resources	238	23.8%	
	Family and friends	249	24.9%	
	Social media	129	12.9%	
	Media (TV, radio, newspapers)	227	22.7%%	

Attitude Question, specific questions related to how participants feel, think, or behave regarding muscle weakness. Response Option, The possible answers. Frequency (N), the number of participants who selected each answer. Percentage (%),the proportion of participants who selected each answer. *p*-value is < 0.05, indicates a significant association between the Attitude responses and demographic variables. *p*-value is > 0.05, no significant difference

When asked which healthcare professional they would first approach for muscle weakness symptoms, neurologists were the most commonly selected option (27.6%, *n* = 276), followed closely by physiotherapists (26.7%, *n* = 267), while 24.8% (*n* = 248) indicated that they would not seek professional assistance. General practitioners were selected by 20.9% (*n* = 209) of respondents. The chi-square analysis was not statistically significant (*p* = 0.07). With respect to willingness to make lifestyle changes, such as increasing exercise or modifying diet to prevent or reduce muscle weakness, the largest proportion of respondents were unsure (39.1%, *n* = 391). In contrast, 32.1% (*n* = 321) reported that they would not be willing to make such changes, while only 28.8% (*n* = 288) expressed willingness to do so. The chi-square analysis showed a statistically significant difference in responses (*p* = 0.04). Lastly, regarding trusted sources of information on muscle weakness the participants indicated that family and friends were the most frequent source (24.9%, *n* = 249) with internet and online resources emerging as second trusted source (23.8%, *n* = 238). Media sources such as television or radio and newspapers had a frequency of (22.7%, *n* = 227) while healthcare providers had a preference rate of (15.7%, *n* = 157). Social media was cited by 12.9% (*n* = 129) of the participants as a source of information on muscle weakness. Chi-square test for significance test showed no statistically significant on differences in trust across information sources (*p* = 0.09).

### Personal experience and impact of muscle weakness on daily life

As captured in Table 5, majority of respondents (67.1%, *n* = 671) reported that neither they nor someone close to them had experienced muscle weakness with only 32.9% (*n* = 329) indicating to have previous personal or indirect experience with the condition. The chi-square analysis demonstrated a statistically significant difference in responses (*p* = 0.02). Among respondents reporting personal experience with muscle weakness or knowing someone affected by the condition, increased reliance on others was the most frequently reported impact on daily life (34.0%, *n* = 322). This was followed by frequent healthcare visits (24.9%, *n* = 240) and then psychological distress (13.4%, *n* = 154) while reduced mobility was cited by 10.9% (*n* = 151) of the participants. Another 13.7% (*n* = 133). The chi-square analysis showed a statistically significant difference in the distribution of reported impacts (*p* = 0.03).

**Table 5:** Participants’ personal experience with muscle weakness

Question	Response Option	Frequency (N)	Percentage (%)	Chi-Square (*p*-value)
Have you or someone close to you experienced muscle weakness?	Yes	329	32.9%	0.02
	No	671	67.1%	
If yes, how has muscle weakness impacted daily life?	Reduced mobility	151	10.9% of "Yes"	0.03
	Increased reliance on others	322	34.0% of "Yes"	
	Frequent healthcare visits	240	24.9% of "Yes"	
	Psychological distress	154	13.4% of "Yes"	
	Never affected my life	133	13.7% of "Yes"	

Response Option,The possible answers.Frequency (N),the number of participants who selected each answer. Percentage (%),the proportion of participants who selected each answer. *p*-value is < 0.05, indicates a significant association between the Personal Experience with Muscle Weakness and demographic variables. *p*-value is > 0.05, no significant difference

## Discussion

This study examined the demographic influences, knowledge, perceptions, attitudes, and personal experiences related to muscle weakness among Saudi adults. The findings provide a nuanced understanding of how individual and societal factors intersect in shaping awareness, beliefs, and behaviors regarding muscle weakness.

### Demographic influences on awareness and knowledge

The results indicated that education showed a borderline association with awareness of muscle weakness, whereas age was not significantly associated with awareness in this sample. Participants with higher educational attainment appeared more likely to recognize muscle weakness as a treatable condition and to identify its causes and symptoms. This finding aligns with research by Al-Essa et al. [3], which highlighted education as a key determinant of health literacy in the Middle East. Although age was not significantly associated with awareness in the present study, previous research has suggested that younger populations may have greater access to digital health resources. The imbalance in knowledge among older participants and those with lower educational levels underscores the need for targeted health education interventions. As suggested by [1, 2] tailored community-based outreach programs can bridge knowledge gaps in underrepresented groups, particularly in areas with cultural stigmas or limited healthcare access.

### Perceptions of muscle weakness severity and challenges

The findings relating to perceptions of muscle weakness suggest a considerable degree of uncertainty regarding personal susceptibility and the potential consequences of the condition as well as the populations most likely to be affected. Interpreted in context, this is an important finding as this uncertainty is a reflection of broader gaps in public understanding of musculoskeletal and neuromuscular health where muscle weakness is generally viewed as a normal consequence of aging rather than a symptom that may indicate underlying disease chronic health conditions [7]. This perception is consistent with evidence indicating that members of the public frequently underestimate the clinical significance of muscle weakness and may fail to recognize its role as an early indicator of functional decline and reduced quality of life [8, 26]. Digging deeper into the existing literature, recent studies have emphasized that muscle weakness is not exclusively an age-related phenomenon but can affect individuals across the lifespan particularly those with chronic diseases or inadequate nutritional status [36]. The observed uncertainty regarding the severity of muscle weakness is also a potential indicator of limited exposure to educational information as the demographic results had indicated. Furthermore, confidence in recognizing signs of muscle weakness appears to coexist with incomplete understanding of the condition which is a phenomenon commonly observed in health literacy research whereby perceived knowledge exceeds actual knowledge. This discrepancy may present the danger of delaying timely healthcare-seeking behavior and reduce opportunities for early intervention.

### Attitudes towards prevention and treatment

The pattern of attitudes that emerged here towards muscle weakness is fundamentally a reflection of a complex interplay between awareness and uncertainty as well as health-related decision-making rather than a uniformly positive or proactive orientation. While many participants acknowledged the importance of seeking medical advice and expressed some willingness to act on symptoms, an important point note here is that the overall distribution suggests that this recognition is not consistently translated into strong behavioral intention. This gap between perceived importance and actionable intent is commonly observed in health behavior research and is well explained by frameworks like the Health Belief Model where perceived severity and susceptibility alone are insufficient to drive behavior in the absence of perceived benefits, cues to action, and reduced barriers to care [15, 17]. In this context, the ambivalence observed in encouraging others to seek help and in willingness to adopt lifestyle modifications is a vital reflection of competing influences from limited confidence in symptom interpretation to competing health priorities to even uncertainty about the effectiveness of interventions. The preference for diverse healthcare entry points including primary care physicians and specialists such as physiotherapists and neurologists further points to an important variability in health system navigation knowledge. The notable proportion of respondents who indicated they would not seek professional help should here be interpreted to imply a potential structural or informational barriers like lack of clarity about appropriate care pathways or underestimation of symptom significance. Similarly, the reliance on informal sources for health information is an indication of a fragmented trust landscape where professional medical sources are not the sole or dominant authority. This aligns with recent literature highlighting the increasing role of digital and social networks in shaping health perceptions, particularly in contexts where health literacy is moderate or uneven [6, 38]. However, such reliance is also a possible factor exposing individuals to misinformation or incomplete guidance and potentially influencing decision-making quality.

### Personal experience with muscle weakness

The findings on personal experience with muscle weakness as emerged here point that direct or indirect exposure to the condition is present in a substantial minority of the population and that this exposure appears to play a key role in shaping how the condition is socially and clinically understood. According to Jung and Song [21], experiential contact with illness is widely recognized in health behavior literature as a critical determinant of risk perception and health-related decision-making and particularly exerting a stronger influence than abstract knowledge alone. Individuals who have observed muscle weakness in themselves or others are potentially more likely to recognize its functional consequences in relation to reduced mobility and increased dependency as well as greater healthcare utilization. In context, these impacts emerge here as reflecting the broader burden of muscle weakness as a condition that affects not only physical capacity but also autonomy and quality of life thus reinforcing its relevance as a public health concern. Further, the prominence of increased reliance on others as a reported consequence shows the social dimension of muscle weakness within which functional decline extends beyond individual health to affect caregiving dynamics and household roles. Similarly, the reported need for frequent healthcare visits suggests that muscle weakness may contribute to increased healthcare system engagement, potentially reflecting both acute episodes and ongoing management of underlying conditions. Although less frequently reported, the findings here show that psychological distress remains an important dimension, aligning with literature that links physical decline to reduced mental well-being and increased vulnerability to anxiety and depression.

### The impact of demographics and perceptions on healthcare responses to muscle weakness in Saudi Arabia

The study’s findings reveal several important interrelationships that offer insight into the awareness and management of muscle weakness. First, demographic factors showed variable associations with awareness levels. Education showed a borderline association with awareness, while age was not significantly associated in this sample. Participants with higher educational attainment appeared more likely to demonstrate better understanding of muscle weakness and related healthcare-seeking behaviors. This increased awareness, in turn, positively affected their attitudes and willingness to seek treatment. This aligns with previous studies showing that education and health literacy may contribute to a more proactive health approach. Additionally, perceptions of this condition was linked to health behaviors. Those who viewed muscle weakness as a severe condition were more inclined to advocate for early medical intervention, a finding consistent with the health belief model [23]. Finally, personal experience with muscle weakness was found to heighten the perceived severity of the condition, as individuals who had encountered it firsthand were more likely to recognize the importance of preventive measures. This suggests that direct exposure to health conditions plays a crucial role in shaping health attitudes.

Based on these findings, several key implications and recommendations emerge. Health education campaigns should focus on populations with lower awareness and lower educational attainment, emphasizing the treatability and prevention of muscle weakness. These groups may benefit from tailored interventions that improve their awareness and attitudes toward the condition. Furthermore, the development of digital health tools, such as culturally relevant online resources, could be an effective strategy for reaching younger demographics who are more likely to engage with digital platforms [11, 12]. Finally, enhancing healthcare access and training, particularly for primary care physicians, is essential to address muscle weakness and related conditions early. By equipping healthcare professionals with the knowledge and tools to diagnose and manage muscle weakness, this could help bridge the gap in early detection and treatment [27].

### Limitations

This study has several limitations. First, the cross-sectional design limits the ability to infer causal relationships between demographic factors and awareness, perceptions, or healthcare-seeking behaviors. Second, the use of convenience sampling and online survey distribution may have introduced selection bias, particularly by favoring individuals with internet access, higher digital literacy, or greater interest in health-related topics. Third, the data were self-reported and may therefore be affected by recall bias or social desirability bias. Fourth, although the questionnaire was developed specifically for this study and pretested for clarity, further psychometric validation may be needed to confirm its reliability and validity across different Saudi population groups. The use of a newly developed questionnaire without extensive psychometric testing may limit measurement accuracy. Finally, some subgroups, including older adults and individuals with lower educational attainment, may have been underrepresented, which may limit the generalizability of the findings.

## Conclusion

This study assessed awareness, perceptions, attitudes, and personal experience related to muscle weakness among Saudi adults. There are mixed levels of understanding among the study population with noticeable gaps in knowledge and variability in not only perceptions of risk and severity but also management. Although many participants demonstrated willingness to seek or recommend medical advice, uncertainty remained in several domain from symptom recognition to preventive actions. Ultimately, strengthening public health education through primary care services and reliable digital platforms present an important opportunity towards improving early recognition and support timely, evidence-based healthcare-seeking behavior.

## Supplementary Information

Supplementary Material 1.

Supplementary Material 2.

## Data Availability

The datasets generated and/or analyzed during the current study are available from the corresponding author upon reasonable request.

## Abbreviations

MG: Myasthenia gravis
SPSS: Statistical Package for the Social Sciences
IRB: Institutional Review Board
