# Supplementary material for: Exploring perceptions, awareness, and healthcare-seeking behaviors related to muscle weakness among Saudi adults: a cross-sectional study
Source: BMC Public Health. 2026 Aug 3;26:3110. doi: 10.1186/s12889-026-28692-4 (PMC13721463; doi:10.1186/s12889-026-28692-4)
Supplement: Supplementary file 1 [file 12889_2026_28692_MOESM1_ESM.docx]

**Supplementary File 1
English-Language Questionnaire**

*Exploring Perceptions, Awareness, and Healthcare-Seeking Behaviors Related to Muscle*

*Weakness Among Saudi Adults: A Cross-Sectional Study*

Instructions for participants: Please read each question carefully and select the answer that best reflects your knowledge, opinion, or experience. Participation is voluntary and responses will be kept confidential.

# Section 1. Demographic Information

1. What is your gender?

☐ Male

☐ Female

2. What is your age group?

 18–29 years

 30–39 years

 40–49 years

 50 years and above

3. What is your highest educational level?

 No formal education

 Primary school

 Secondary school

 Diploma

 Bachelor's degree

 Postgraduate degree

4. What is your region of residence in Saudi Arabia?

☐ Central

☐ Eastern

☐ Western

☐ Northern

☐ Southern

5. What is your current occupation?

☐ Student

☐ Employed

☐ Unemployed

☐ Retired

# Section 2. Knowledge of Muscle Weakness

6. Have you ever heard of muscle weakness as a medical condition?

☐ Yes

☐ No

7. Which of the following do you think are common causes of muscle weakness? Please select all that apply.

☐ Aging

☐ Chronic diseases such as diabetes or heart disease

☐ Lack of physical activity

☐ Malnutrition

☐ Injuries or trauma

8. What do you think are common symptoms of muscle weakness? Please select all that apply.

☐ Difficulty in moving or lifting objects

☐ Muscle fatigue after minimal effort

☐ Reduced ability to perform daily tasks

☐ Pain or discomfort in muscles

☐ Frequent falls or instability

9. Do you believe muscle weakness is a treatable condition?

☐ Yes

☐ No

☐ Not sure

10. Which treatments do you think could be effective for managing muscle weakness? Please select all that apply.

☐ Physical therapy/exercise

☐ Medications

☐ Surgery

☐ Nutritional supplements

☐ Lifestyle modifications

# Section 3. Perceptions and Awareness of Muscle Weakness

11. How likely do you think you are to develop muscle weakness at some point in your life?

☐ Very unlikely

☐ Unlikely

☐ Neutral

☐ Likely

☐ Very likely

12. How severe do you think the impact of muscle weakness can be on a person's life?

☐ Not severe at all

☐ Slightly severe

☐ Moderately severe

☐ Very severe

☐ Extremely severe

13. In your opinion, what is the most challenging aspect of living with muscle weakness?

☐ Physical limitations

☐ Pain and discomfort

☐ Reduced independence

☐ Psychological impact such as stress or anxiety

☐ Social isolation

14. Do you believe muscle weakness only affects older adults?

☐ Yes

☐ No

☐ Not sure

15. How confident are you that you can recognize the signs of muscle weakness in yourself or others?

☐ Not confident

☐ Slightly confident

☐ Moderately confident

☐ Very confident

☐ Extremely confident

# Section 4. Attitudes Toward Muscle Weakness and Treatment

16. How important do you think it is to seek medical advice for symptoms of muscle weakness?

☐ Not important

☐ Slightly important

☐ Moderately important

☐ Very important

☐ Extremely important

17. How likely are you to encourage someone with muscle weakness symptoms to seek medical help?

☐ Very unlikely

☐ Unlikely

☐ Neutral

☐ Likely

☐ Very likely

18. What kind of healthcare professional would you first approach for muscle weakness symptoms?

 General practitioner

 Physiotherapist

 Neurologist

 None

19. Would you be willing to make lifestyle changes, such as increased exercise or diet modification, to prevent or reduce muscle weakness?

☐ Yes

☐ No

☐ Not sure

20. What sources do you trust the most for information on muscle weakness?

☐ Healthcare providers

☐ Internet/online resources

☐ Family and friends

☐ Media such as TV, radio, or newspapers

# Section 5. Personal Experience and Impact on Daily Life

21. Have you or someone close to you experienced muscle weakness?

☐ Yes

☐ No

22. If yes, how has muscle weakness impacted daily life? Please select the main impact.

☐Reduced mobility

☐Increased reliance on others

☐Frequent healthcare visits

☐Psychological distress

☐Never affected my life

# Scoring Note

# For data analysis purposes, Likert-scale responses were coded numerically, with higher scores indicating greater levels of agreement, confidence, importance, severity, or likelihood. Response categories were coded according to the number of options available for each item, with the lowest response assigned the lowest score and the highest response assigned the highest score.
